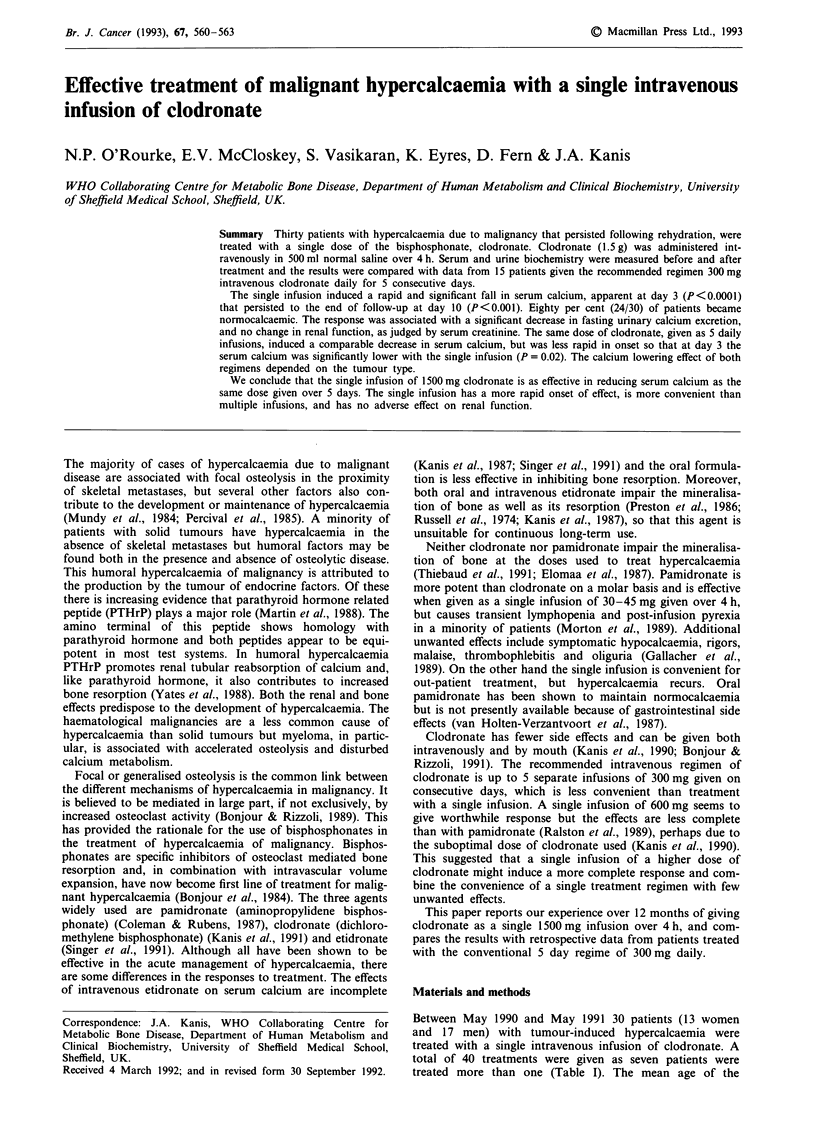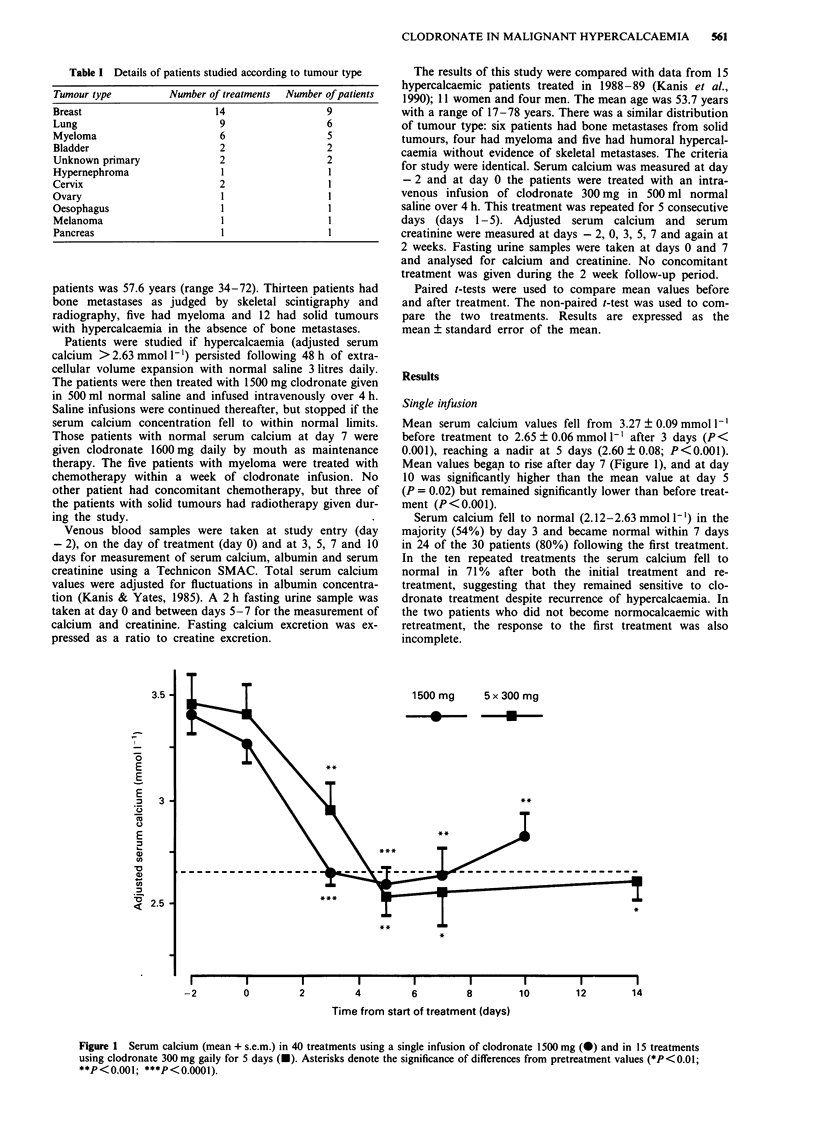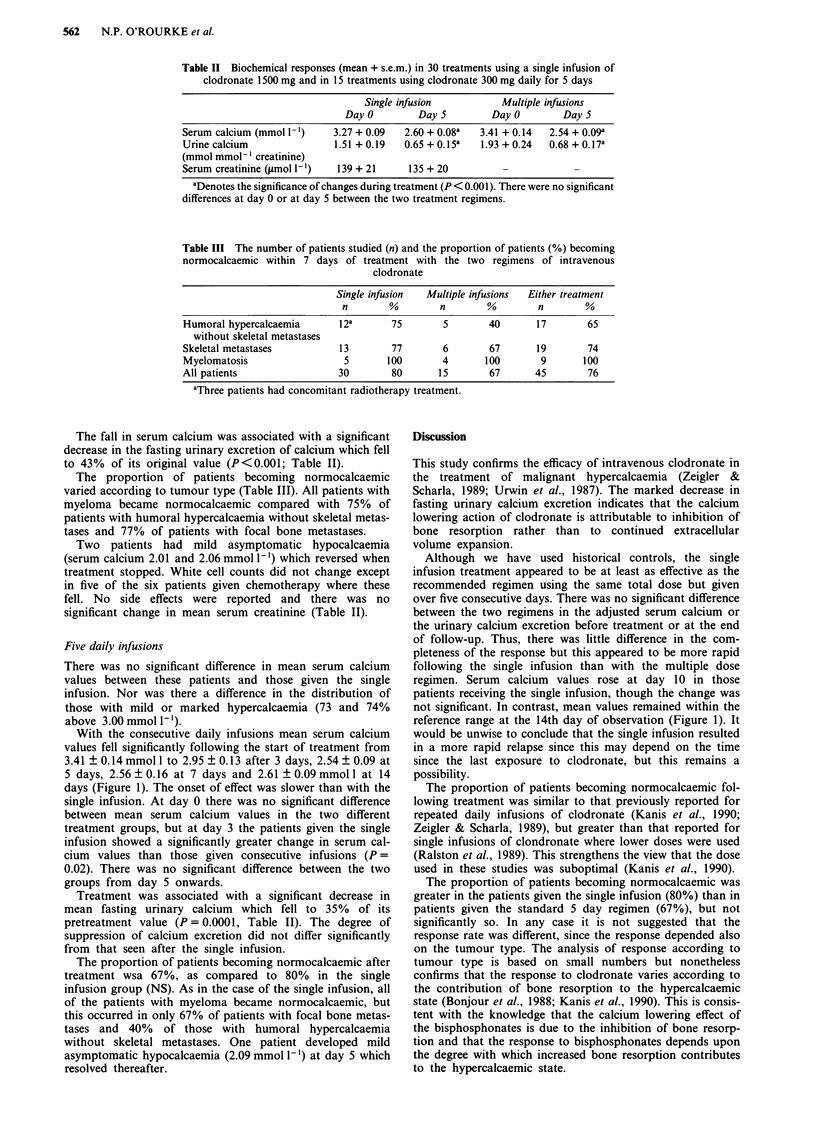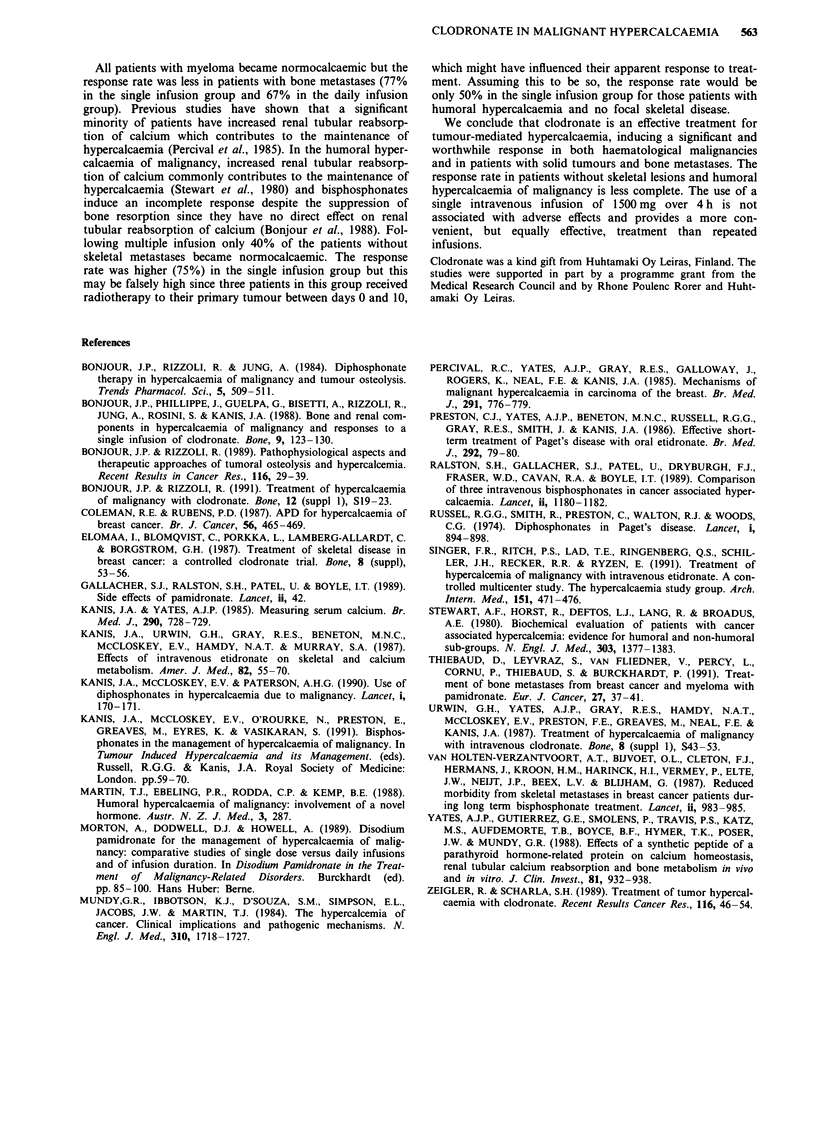# Effective treatment of malignant hypercalcaemia with a single intravenous infusion of clodronate.

**DOI:** 10.1038/bjc.1993.102

**Published:** 1993-03

**Authors:** N. P. O'Rourke, E. V. McCloskey, S. Vasikaran, K. Eyres, D. Fern, J. A. Kanis

**Affiliations:** Department of Human Metabolism and Clinical Biochemistry, University of Sheffield Medical School, UK.

## Abstract

Thirty patients with hypercalcaemia due to malignancy that persisted following rehydration, were treated with a single dose of the bisphosphonate, clodronate. Clodronate (1.5 g) was administered intravenously in 500 ml normal saline over 4 h. Serum and urine biochemistry were measured before and after treatment and the results were compared with data from 15 patients given the recommended regimen 300 mg intravenous clodronate daily for 5 consecutive days. The single infusion induced a rapid and significant fall in serum calcium, apparent at day 3 (P < 0.0001) that persisted to the end of follow-up at day 10 (P < 0.001). Eighty per cent (24/30) of patients became normocalcaemic. The response was associated with a significant decrease in fasting urinary calcium excretion, and no change in renal function, as judged by serum creatinine. The same dose of clodronate, given as 5 daily infusions, induced a comparable decrease in serum calcium, but was less rapid in onset so that at day 3 the serum calcium was significantly lower with the single infusion (P = 0.02). The calcium lowering effect of both regimens depended on the tumour type. We conclude that the single infusion of 1500 mg clodronate is as effective in reducing serum calcium as the same dose given over 5 days. The single infusion has a more rapid onset of effect, is more convenient than multiple infusions, and has no adverse effect on renal function.


Br. J. Cancer (1993), 67, 560-563 © Macmillan Press Ltd., 1993

### Effective treatment of malignant hypercalcaemia with a single intravenous infusion of clodronate

N.P. O'Rourke, E.V. McCloskey, S. Vasikaran, K. Eyres, D. Fern & J.A. Kanis

*WHO Collaborating Centre for Metabolic Bone Disease, Department of Human Metabolism and Clinical Biochemistry, University of Sheffield Medical School, Sheffield, UK.*

**Summary** Thirty patients with hypercalcaemia due to malignancy that persisted following rehydration, were treated with a single dose of the bisphosphonate, clodronate. Clodronate (1.5 g) was administered intravenously in 500 ml normal saline over 4 h. Serum and urine biochemistry were measured before and after treatment and the results were compared with data from 15 patients given the recommended regimen 300 mg intravenous clodronate daily for 5 consecutive days.

The single infusion induced a rapid and significant fall in serum calcium, apparent at day 3 ($P<0.0001$) that persisted to the end of follow-up at day 10 ($P<0.001$). Eighty per cent (24/30) of patients became normocalcaemic. The response was associated with a significant decrease in fasting urinary calcium excretion, and no change in renal function, as judged by serum creatinine. The same dose of clodronate, given as 5 daily infusions, induced a comparable decrease in serum calcium, but was less rapid in onset so that at day 3 the serum calcium was significantly lower with the single infusion ($P = 0.02$). The calcium lowering effect of both regimens depended on the tumour type.

We conclude that the single infusion of 1500 mg clodronate is as effective in reducing serum calcium as the same dose given over 5 days. The single infusion has a more rapid onset of effect, is more convenient than multiple infusions, and has no adverse effect on renal function.

The majority of cases of hypercalcaemia due to malignant disease are associated with focal osteolysis in the proximity of skeletal metastases, but several other factors also contribute to the development or maintenance of hypercalcaemia (Mundy *et al.*, 1984; Percival *et al.*, 1985). A minority of patients with solid tumours have hypercalcaemia in the absence of skeletal metastases but humoral factors may be found both in the presence and absence of osteolytic disease. This humoral hypercalcaemia of malignancy is attributed to the production by the tumour of endocrine factors. Of these there is increasing evidence that parathyroid hormone related peptide (PTHrP) plays a major role (Martin *et al.*, 1988). The amino terminal of this peptide shows homology with parathyroid hormone and both peptides appear to be equipotent in most test systems. In humoral hypercalcaemia PTHrP promotes renal tubular reabsorption of calcium and, like parathyroid hormone, it also contributes to increased bone resorption (Yates *et al.*, 1988). Both the renal and bone effects predispose to the development of hypercalcaemia. The haematological malignancies are a less common cause of hypercalcaemia than solid tumours but myeloma, in particular, is associated with accelerated osteolysis and disturbed calcium metabolism.

Focal or generalised osteolysis is the common link between the different mechanisms of hypercalcaemia in malignancy. It is believed to be mediated in large part, if not exclusively, by increased osteoclast activity (Bonjour & Rizzoli, 1989). This has provided the rationale for the use of bisphosphonates in the treatment of hypercalcaemia of malignancy. Bisphosphonates are specific inhibitors of osteoclast mediated bone resorption and, in combination with intravascular volume expansion, have now become first line of treatment for malignant hypercalcaemia (Bonjour *et al.*, 1984). The three agents widely used are pamidronate (aminopropylidene bisphosphonate) (Coleman & Rubens, 1987), clodronate (dichloromethylene bisphosphonate) (Kanis *et al.*, 1991) and etidronate (Singer *et al.*, 1991). Although all have been shown to be effective in the acute management of hypercalcaemia, there are some differences in the responses to treatment. The effects of intravenous etidronate on serum calcium are incomplete (Kanis *et al.*, 1987; Singer *et al.*, 1991) and the oral formulation is less effective in inhibiting bone resorption. Moreover, both oral and intravenous etidronate impair the mineralisation of bone as well as its resorption (Preston *et al.*, 1986; Russell *et al.*, 1974; Kanis *et al.*, 1987), so that this agent is unsuitable for continuous long-term use.

Neither clodronate nor pamidronate impair the mineralisation of bone at the doses used to treat hypercalcaemia (Thiebaud *et al.*, 1991; Elomaa *et al.*, 1987). Pamidronate is more potent than clodronate on a molar basis and is effective when given as a single infusion of 30-45 mg given over 4 h, but causes transient lymphopenia and post-infusion pyrexia in a minority of patients (Morton *et al.*, 1989). Additional unwanted effects include symptomatic hypocalcaemia, rigors, malaise, thrombophlebitis and oliguria (Gallacher *et al.*, 1989). On the other hand the single infusion is convenient for out-patient treatment, but hypercalcaemia recurs. Oral pamidronate has been shown to maintain normocalcaemia but is not presently available because of gastrointestinal side effects (van Holten-Verzantvoort *et al.*, 1987).

Clodronate has fewer side effects and can be given both intravenously and by mouth (Kanis *et al.*, 1990; Bonjour & Rizzoli, 1991). The recommended intravenous regimen of clodronate is up to 5 separate infusions of 300 mg given on consecutive days, which is less convenient than treatment with a single infusion. A single infusion of 600 mg seems to give worthwhile response but the effects are less complete than with pamidronate (Ralston *et al.*, 1989), perhaps due to the suboptimal dose of clodronate used (Kanis *et al.*, 1990). This suggested that a single infusion of a higher dose of clodronate might induce a more complete response and combine the convenience of a single treatment regimen with few unwanted effects.

This paper reports our experience over 12 months of giving clodronate as a single 1500 mg infusion over 4 h, and compares the results with retrospective data from patients treated with the conventional 5 day regime of 300 mg daily.

### Materials and methods

Between May 1990 and May 1991 30 patients (13 women and 17 men) with tumour-induced hypercalcaemia were treated with a single intravenous infusion of clodronate. A total of 40 treatments were given as seven patients were treated more than one (Table I). The mean age of the

Correspondence: J.A. Kanis, WHO Collaborating Centre for Metabolic Bone Disease, Department of Human Metabolism and Clinical Biochemistry, University of Sheffield Medical School, Sheffield, UK.
Received 4 March 1992; and in revised form 30 September 1992.

**Table I** Details of patients studied according to tumour type

| *Tumour type* | *Number of treatments* | *Number of patients* |
|---|---|---|
| Breast | 14 | 9 |
| Lung | 9 | 6 |
| Myeloma | 6 | 5 |
| Bladder | 2 | 2 |
| Unknown primary | 2 | 2 |
| Hypernephroma | 1 | 1 |
| Cervix | 2 | 1 |
| Ovary | 1 | 1 |
| Oesophagus | 1 | 1 |
| Melanoma | 1 | 1 |
| Pancreas | 1 | 1 |

patients was 57.6 years (range 34–72). Thirteen patients had bone metastases as judged by skeletal scintigraphy and radiography, five had myeloma and 12 had solid tumours with hypercalcaemia in the absence of bone metastases.

Patients were studied if hypercalcaemia (adjusted serum calcium $>2.63$ mmol $l^{-1}$) persisted following 48 h of extracellular volume expansion with normal saline 3 litres daily. The patients were then treated with 1500 mg clodronate given in 500 ml normal saline and infused intravenously over 4 h. Saline infusions were continued thereafter, but stopped if the serum calcium concentration fell to within normal limits. Those patients with normal serum calcium at day 7 were given clodronate 1600 mg daily by mouth as maintenance therapy. The five patients with myeloma were treated with chemotherapy within a week of clodronate infusion. No other patient had concomitant chemotherapy, but three of the patients with solid tumours had radiotherapy given during the study.

Venous blood samples were taken at study entry (day – 2), on the day of treatment (day 0) and at 3, 5, 7 and 10 days for measurement of serum calcium, albumin and serum creatinine using a Technicon SMAC. Total serum calcium values were adjusted for fluctuations in albumin concentration (Kanis & Yates, 1985). A 2 h fasting urine sample was taken at day 0 and between days 5–7 for the measurement of calcium and creatinine. Fasting calcium excretion was expressed as a ratio to creatine excretion.

The results of this study were compared with data from 15 hypercalcaemic patients treated in 1988–89 (Kanis *et al.*, 1990); 11 women and four men. The mean age was 53.7 years with a range of 17–78 years. There was a similar distribution of tumour type: six patients had bone metastases from solid tumours, four had myeloma and five had humoral hypercalcaemia without evidence of skeletal metastases. The criteria for study were identical. Serum calcium was measured at day – 2 and at day 0 the patients were treated with an intravenous infusion of clodronate 300 mg in 500 ml normal saline over 4 h. This treatment was repeated for 5 consecutive days (days 1–5). Adjusted serum calcium and serum creatinine were measured at days – 2, 0, 3, 5, 7 and again at 2 weeks. Fasting urine samples were taken at days 0 and 7 and analysed for calcium and creatinine. No concomitant treatment was given during the 2 week follow-up period.

Paired *t*-tests were used to compare mean values before and after treatment. The non-paired *t*-test was used to compare the two treatments. Results are expressed as the mean ± standard error of the mean.

### Results

### *Single infusion*

Mean serum calcium values fell from $3.27 \pm 0.09$ mmol $l^{-1}$ before treatment to $2.65 \pm 0.06$ mmol $l^{-1}$ after 3 days ($P <$ 0.001), reaching a nadir at 5 days ($2.60 \pm 0.08$; $P < 0.001$). Mean values began to rise after day 7 (Figure 1), and at day 10 was significantly higher than the mean value at day 5 ($P = 0.02$) but remained significantly lower than before treatment ($P < 0.001$).

Serum calcium fell to normal (2.12–2.63 mmol $l^{-1}$) in the majority (54%) by day 3 and became normal within 7 days in 24 of the 30 patients (80%) following the first treatment. In the ten repeated treatments the serum calcium fell to normal in 71% after both the initial treatment and retreatment, suggesting that they remained sensitive to clodronate treatment despite recurrence of hypercalcaemia. In the two patients who did not become normocalcaemic with retreatment, the response to the first treatment was also incomplete.

**Figure 1** Serum calcium (mean + s.e.m.) in 40 treatments using a single infusion of clodronate 1500 mg (●) and in 15 treatments using clodronate 300 mg gaily for 5 days (■). Asterisks denote the significance of differences from pretreatment values (*$P<0.01$; **$P<0.001$; ***$P<0.0001$).

**Table II** Biochemical responses (mean + s.e.m.) in 30 treatments using a single infusion of clodronate 1500 mg and in 15 treatments using clodronate 300 mg daily for 5 days

| | *Single infusion* | | *Multiple infusions* | |
|---|---|---|---|---|
| | *Day 0* | *Day 5* | *Day 0* | *Day 5* |
| Serum calcium (mmol $l^{-1}$) | 3.27 + 0.09 | 2.60 + 0.08[a] | 3.41 + 0.14 | 2.54 + 0.09[a] |
| Urine calcium (mmol $mmol^{-1}$ creatinine) | 1.51 + 0.19 | 0.65 + 0.15[a] | 1.93 + 0.24 | 0.68 + 0.17[a] |
| Serum creatinine (μmol $l^{-1}$) | 139 + 21 | 135 + 20 | – | – |

[a]Denotes the significance of changes during treatment ($P < 0.001$). There were no significant differences at day 0 or at day 5 between the two treatment regimens.

**Table III** The number of patients studied (*n*) and the proportion of patients (%) becoming normocalcaemic within 7 days of treatment with the two regimens of intravenous clodronate

| | *Single infusion* | | *Multiple infusions* | | *Either treatment* | |
|---|---|---|---|---|---|---|
| | *n* | % | *n* | % | *n* | % |
| Humoral hypercalcaemia without skeletal metastases | 12[a] | 75 | 5 | 40 | 17 | 65 |
| Skeletal metastases | 13 | 77 | 6 | 67 | 19 | 74 |
| Myelomatosis | 5 | 100 | 4 | 100 | 9 | 100 |
| All patients | 30 | 80 | 15 | 67 | 45 | 76 |

[a]Three patients had concomitant radiotherapy treatment.

The fall in serum calcium was associated with a significant decrease in the fasting urinary excretion of calcium which fell to 43% of its original value ($P<0.001$; Table II).

The proportion of patients becoming normocalcaemic varied according to tumour type (Table III). All patients with myeloma became normocalcaemic compared with 75% of patients with humoral hypercalcaemia without skeletal metastases and 77% of patients with focal bone metastases.

Two patients had mild asymptomatic hypocalcaemia (serum calcium 2.01 and 2.06 mmol $l^{-1}$) which reversed when treatment stopped. White cell counts did not change except in five of the six patients given chemotherapy where these fell. No side effects were reported and there was no significant change in mean serum creatinine (Table II).

*Five daily infusions*

There was no significant difference in mean serum calcium values between these patients and those given the single infusion. Nor was there a difference in the distribution of those with mild or marked hypercalcaemia (73 and 74% above 3.00 mmol $l^{-1}$).

With the consecutive daily infusions mean serum calcium values fell significantly following the start of treatment from 3.41 ± 0.14 mmol l to 2.95 ± 0.13 after 3 days, 2.54 ± 0.09 at 5 days, 2.56 ± 0.16 at 7 days and 2.61 ± 0.09 mmol l at 14 days (Figure 1). The onset of effect was slower than with the single infusion. At day 0 there was no significant difference between mean serum calcium values in the two different treatment groups, but at day 3 the patients given the single infusion showed a significantly greater change in serum calcium values than those given consecutive infusions ($P = 0.02$). There was no significant difference between the two groups from day 5 onwards.

Treatment was associated with a significant decrease in mean fasting urinary calcium which fell to 35% of its pretreatment value ($P = 0.0001$, Table II). The degree of suppression of calcium excretion did not differ significantly from that seen after the single infusion.

The proportion of patients becoming normocalcaemic after treatment wsa 67%, as compared to 80% in the single infusion group (NS). As in the case of the single infusion, all of the patients with myeloma became normocalcaemic, but this occurred in only 67% of patients with focal bone metastases and 40% of those with humoral hypercalcaemia without skeletal metastases. One patient developed mild asymptomatic hypocalcaemia (2.09 mmol $l^{-1}$) at day 5 which resolved thereafter.

### Discussion

This study confirms the efficacy of intravenous clodronate in the treatment of malignant hypercalcaemia (Zeigler & Scharla, 1989; Urwin *et al.*, 1987). The marked decrease in fasting urinary calcium excretion indicates that the calcium lowering action of clodronate is attributable to inhibition of bone resorption rather than to continued extracellular volume expansion.

Although we have used historical controls, the single infusion treatment appeared to be at least as effective as the recommended regimen using the same total dose but given over five consecutive days. There was no significant difference between the two regimens in the adjusted serum calcium or the urinary calcium excretion before treatment or at the end of follow-up. Thus, there was little difference in the completeness of the response but this appeared to be more rapid following the single infusion than with the multiple dose regimen. Serum calcium values rose at day 10 in those patients receiving the single infusion, though the change was not significant. In contrast, mean values remained within the reference range at the 14th day of observation (Figure 1). It would be unwise to conclude that the single infusion resulted in a more rapid relapse since this may depend on the time since the last exposure to clodronate, but this remains a possibility.

The proportion of patients becoming normocalcaemic following treatment was similar to that previously reported for repeated daily infusions of clodronate (Kanis *et al.*, 1990; Zeigler & Scharla, 1989), but greater than that reported for single infusions of clondronate where lower doses were used (Ralston *et al.*, 1989). This strengthens the view that the dose used in these studies was suboptimal (Kanis *et al.*, 1990).

The proportion of patients becoming normocalcaemic was greater in the patients given the single infusion (80%) than in patients given the standard 5 day regimen (67%), but not significantly so. In any case it is not suggested that the response rate was different, since the response depended also on the tumour type. The analysis of response according to tumour type is based on small numbers but nonetheless confirms that the response to clodronate varies according to the contribution of bone resorption to the hypercalcaemic state (Bonjour *et al.*, 1988; Kanis *et al.*, 1990). This is consistent with the knowledge that the calcium lowering effect of the bisphosphonates is due to the inhibition of bone resorption and that the response to bisphosphonates depends upon the degree with which increased bone resorption contributes to the hypercalcaemic state.

All patients with myeloma became normocalcaemic but the response rate was less in patients with bone metastases (77% in the single infusion group and 67% in the daily infusion group). Previous studies have shown that a significant minority of patients have increased renal tubular reabsorption of calcium which contributes to the maintenance of hypercalcaemia (Percival *et al.*, 1985). In the humoral hypercalcaemia of malignancy, increased renal tubular reabsorption of calcium commonly contributes to the maintenance of hypercalcaemia (Stewart *et al.*, 1980) and bisphosphonates induce an incomplete response despite the suppression of bone resorption since they have no direct effect on renal tubular reabsorption of calcium (Bonjour *et al.*, 1988). Following multiple infusion only 40% of the patients without skeletal metastases became normocalcaemic. The response rate was higher (75%) in the single infusion group but this may be falsely high since three patients in this group received radiotherapy to their primary tumour between days 0 and 10, which might have influenced their apparent response to treatment. Assuming this to be so, the response rate would be only 50% in the single infusion group for those patients with humoral hypercalcaemia and no focal skeletal disease.

We conclude that clodronate is an effective treatment for tumour-mediated hypercalcaemia, inducing a significant and worthwhile response in both haematological malignancies and in patients with solid tumours and bone metastases. The response rate in patients without skeletal lesions and humoral hypercalcaemia of malignancy is less complete. The use of a single intravenous infusion of 1500 mg over 4 h is not associated with adverse effects and provides a more convenient, but equally effective, treatment than repeated infusions.

Clodronate was a kind gift from Huhtamaki Oy Leiras, Finland. The studies were supported in part by a programme grant from the Medical Research Council and by Rhone Poulenc Rorer and Huhtamaki Oy Leiras.

### References

BONJOUR, J.P., RIZZOLI, R. & JUNG, A. (1984). Diphosphonate therapy in hypercalcaemia of malignancy and tumour osteolysis. *Trends Pharmacol. Sci.*, **5**, 509–511.

BONJOUR, J.P., PHILLIPPE, J., GUELPA, G., BISETTI, A., RIZZOLI, R., JUNG, A., ROSINI, S. & KANIS, J.A. (1988). Bone and renal components in hypercalcaemia of malignancy and responses to a single infusion of clodronate. *Bone*, **9**, 123–130.

BONJOUR, J.P. & RIZZOLI, R. (1989). Pathophysiological aspects and therapeutic approaches of tumoral osteolysis and hypercalcemia. *Recent Results in Cancer Res.*, **116**, 29–39.

BONJOUR, J.P. & RIZZOLI, R. (1991). Treatment of hypercalcaemia of malignancy with clodronate. *Bone*, **12** (suppl 1), S19–23.

COLEMAN, R.E. & RUBENS, P.D. (1987). APD for hypercalcaemia of breast cancer. *Br. J. Cancer*, **56**, 465–469.

ELOMAA, I., BLOMQVIST, C., PORKKA, L., LAMBERG-ALLARDT, C. & BORGSTROM, G.H. (1987). Treatment of skeletal disease in breast cancer: a controlled clodronate trial. *Bone*, **8** (suppl), 53–56.

GALLACHER, S.J., RALSTON, S.H., PATEL, U. & BOYLE, I.T. (1989). Side effects of pamidronate. *Lancet*, **ii**, 42.

KANIS, J.A. & YATES, A.J.P. (1985). Measuring serum calcium. *Br. Med. J.*, **290**, 728–729.

KANIS, J.A., URWIN, G.H., GRAY, R.E.S., BENETON, M.N.C., MCCLOSKEY, E.V., HAMDY, N.A.T. & MURRAY, S.A. (1987). Effects of intravenous etidronate on skeletal and calcium metabolism. *Amer. J. Med.*, **82**, 55–70.

KANIS, J.A., MCCLOSKEY, E.V. & PATERSON, A.H.G. (1990). Use of diphosphonates in hypercalcaemia due to malignancy. *Lancet*, **i**, 170–171.

KANIS, J.A., MCCLOSKEY, E.V., O'ROURKE, N., PRESTON, E., GREAVES, M., EYRES, K. & VASIKARAN, S. (1991). Bisphosphonates in the management of hypercalcaemia of malignancy. In *Tumour Induced Hypercalcaemia and its Management*. (eds). Russell, R.G.G. & Kanis, J.A. Royal Society of Medicine: London. pp.59–70.

MARTIN, T.J., EBELING, P.R., RODDA, C.P. & KEMP, B.E. (1988). Humoral hypercalcaemia of malignancy: involvement of a novel hormone. *Austr. N. Z. J. Med.*, **3**, 287.

MORTON, A., DODWELL, D.J. & HOWELL, A. (1989). Disodium pamidronate for the management of hypercalcaemia of malignancy: comparative studies of single dose versus daily infusions and of infusion duration. In *Disodium Pamidronate in the Treatment of Malignancy-Related Disorders*. Burckhardt (ed). pp. 85–100. Hans Huber: Berne.

MUNDY,G.R., IBBOTSON, K.J., D'SOUZA, S.M., SIMPSON, E.L., JACOBS, J.W. & MARTIN, T.J. (1984). The hypercalcemia of cancer. Clinical implications and pathogenic mechanisms. *N. Engl. J. Med.*, **310**, 1718–1727.

PERCIVAL, R.C., YATES, A.J.P., GRAY, R.E.S., GALLOWAY, J., ROGERS, K., NEAL, F.E. & KANIS, J.A. (1985). Mechanisms of malignant hypercalcaemia in carcinoma of the breast. *Br. Med. J.*, **291**, 776–779.

PRESTON, C.J., YATES, A.J.P., BENETON, M.N.C., RUSSELL, R.G.G., GRAY, R.E.S., SMITH, J. & KANIS, J.A. (1986). Effective short-term treatment of Paget's disease with oral etidronate. *Br. Med. J.*, **292**, 79–80.

RALSTON, S.H., GALLACHER, S.J., PATEL, U., DRYBURGH, F.J., FRASER, W.D., CAVAN, R.A. & BOYLE, I.T. (1989). Comparison of three intravenous bisphosphonates in cancer associated hypercalcaemia. *Lancet*, **ii**, 1180–1182.

RUSSEL, R.G.G., SMITH, R., PRESTON, C., WALTON, R.J. & WOODS, C.G. (1974). Diphosphonates in Paget's disease. *Lancet*, **i**, 894–898.

SINGER, F.R., RITCH, P.S., LAD, T.E., RINGENBERG, Q.S., SCHILLER, J.H., RECKER, R.R. & RYZEN, E. (1991). Treatment of hypercalcemia of malignancy with intravenous etidronate. A controlled multicenter study. The hypercalcaemia study group. *Arch. Intern. Med.*, **151**, 471–476.

STEWART, A.F., HORST, R., DEFTOS, L.J., LANG, R. & BROADUS, A.E. (1980). Biochemical evaluation of patients with cancer associated hypercalcemia: evidence for humoral and non-humoral sub-groups. *N. Engl. J. Med.*, **303**, 1377–1383.

THIEBAUD, D., LEYVRAZ, S., VAN FLIEDNER, V., PERCY, L., CORNU, P., THIEBAUD, S. & BURCKHARDT, P. (1991). Treatment of bone metastases from breast cancer and myeloma with pamidronate. *Eur. J. Cancer*, **27**, 37–41.

URWIN, G.H., YATES, A.J.P., GRAY, R.E.S., HAMDY, N.A.T., MCCLOSKEY, E.V., PRESTON, F.E., GREAVES, M., NEAL, F.E. & KANIS, J.A. (1987). Treatment of hypercalcaemia of malignancy with intravenous clodronate. *Bone*, **8** (suppl 1), S43–53.

VAN HOLTEN-VERZANTVOORT, A.T., BIJVOET, O.L., CLETON, F.J., HERMANS, J., KROON, H.M., HARINCK, H.I., VERMEY, P., ELTE, J.W., NEIJT, J.P., BEEX, L.V. & BLIJHAM, G. (1987). Reduced morbidity from skeletal metastases in breast cancer patients during long term bisphosphonate treatment. *Lancet*, **ii**, 983–985.

YATES, A.J.P., GUTIERREZ, G.E., SMOLENS, P., TRAVIS, P.S., KATZ, M.S., AUFDEMORTE, T.B., BOYCE, B.F., HYMER, T.K., POSER, J.W. & MUNDY, G.R. (1988). Effects of a synthetic peptide of a parathyroid hormone-related protein on calcium homeostasis, renal tubular calcium reabsorption and bone metabolism *in vivo* and *in vitro*. *J. Clin. Invest.*, **81**, 932–938.

ZEIGLER, R. & SCHARLA, S.H. (1989). Treatment of tumor hypercalcaemia with clodronate. *Recent Results Cancer Res.*, **116**, 46–54.